# Simulation-based interdisciplinary education improves intern attitudes and outlook toward colleagues in other disciplines

**DOI:** 10.1186/s12909-019-1700-1

**Published:** 2019-07-24

**Authors:** Mark J. Bullard, Sean M. Fox, Catherine M. Wares, Alan C. Heffner, Casey Stephens, Laura Rossi

**Affiliations:** 1 Department of Emergency Medicine, Carolinas Medical Center, Carolinas Simulation Center, Atrium Health, 1000 Blythe Blvd., 3rd Floor MEB, Boston, Massachusetts 28203 USA; 2 Department of Emergency Medicine, Carolinas Medical Center, Atrium Health, Boston, Massachusetts USA; 3 Department of Emergency Medicine, Department of Internal Medicine, Division of Critical Care, Carolinas Medical Center, Atrium Health, Boston, Massachusetts USA; 4 Center for Outcomes Research and Evaluation, Atrium Health, Boston, Massachusetts USA; 5 Simmons University, College of Natural and Behavioral Sciences, MGH Institute of Health Professions, MGH Department of Orthopedic Surgery Quality and Safety Program, Boston, Massachusetts USA

## Abstract

**Background:**

Cooperative interdisciplinary patient care is a modern healthcare necessity. While various medical and surgical disciplines have independent educational requirements, a system-wide simulation-based curriculum composed of different disciplines provides a unique forum to observe the effect of interdisciplinary simulation-based education (IDSE). Our hypothesis: IDSE positively affects intern outlook and attitudes towards other medical disciplines.

**Methods:**

Using an established interdisciplinary simulation curriculum designed for first year interns, we explored the relative effect of IDSE on between-discipline intern attitudes in a convergent, parallel, mixed-methods study. Data sources included novel pre-post anonymous survey measurements (10-point Likert scale), focus groups, direct observations, and reflective field notes. This quasi-experimental pilot study was conducted at an academic, tertiary care medical center with two cohorts of interns: one exposed to IDSE and one exposed to an independent within-discipline simulation curriculum.

**Results:**

IDSE exposed interns demonstrated statistically significant improvements when comparing mean pre-test and post-test score differences in five of seven areas: perceived interdisciplinary collegiality ($\overline{x}$ = 0.855; *p* = 0.0002), respect (*x̅* = 0.436; *p* = 0.0312), work interactions ($\overline{x}$ = 0.691; *p* = 0.0069), perceived interdisciplinary attitudes (*x̅* = 0.764; *p* = 0.0031), and comfort in interdisciplinary learning (*x̅* = 1.164; *p* < 0.0001). There were no changes in interdisciplinary viewpoints observed among non-IDSE interns. IDSE interns were comfortable when learning with interns of different disciplines and believed others viewed their discipline positively compared to non-IDSE interns. Qualitative data uncovered the following themes related to the impact of IDSE including: 1) Relationship building, 2) Communication openness, 3) Attitude shifting, and 4) Enhanced learner experience.

**Conclusions:**

IDSE positively influenced intern outlook on and attitudes towards other medical disciplines. This unique learning environment provided interns an opportunity to learn clinical case management while learning about, from, and with each other; subsequently breaking traditional discipline-specific stereotypes and improving interdisciplinary relations. Future explicit focus on IDSE offers opportunity to improve interdisciplinary interactions and patient care.

## Background

Medical simulation improves knowledge and skills acquisition while creating an educational method with a high level of learner satisfaction [1]. Simulation is a unique educational modality because it alleviates educational constraints of case availability, unifies learner exposure to critical thinking, hones procedural skills prior to performance on patients, allows for exposure to complex ethical and spiritual issues, and refines important interpersonal professional skillsets [2, 3]. Additionally, simulation education provides a forum for interprofessional education: to learn about, from and with each other [4].

Cooperative interdisciplinary patient care is a modern healthcare necessity. It has been suggested that “… the educational system [is] one of the main determinants of interprofessional collaborative practice, because it represents the principal lever for promoting collaborative values among future healthcare professionals” [5]. Anecdotal accounts of adversarial interactions in clinical practice are often influenced by a lack of understanding or familiarity with others’ perspectives. As such, interprofessional education (IPE) has been increasingly highlighted as an opportunity to improve professional development and patient outcomes.

While IPE is technically defined as occurring when students from two or more professions learn about, from, and with each other to enable effective collaboration and improve health outcomes [4], we propose that interdisciplinary education, with students from two or more disciplines within a specific profession, may be viewed through the same lens and abide by the same frameworks. Simulation-based IPE has demonstrated positive effects for interprofessional collaboration [6], teamwork [7, 8], communication skills [8], and even clinical outcomes as measured within the simulated setting [7]. There remains limited research addressing whether interprofessional education affects attitudes between professions [9]. Even more limited is specific research pertaining to whether IPE within the simulation-based learning environment affects interprofessional attitudes, prejudices, and stereotypes [6].

The purpose of this convergent, parallel, mixed methods pilot study was to explore the impact of interdisciplinary simulation-based education (IDSE) on between-discipline intern outlook and attitudes. Specifically, viewpoints of medical interns exposed to an existing longitudinal IDSE curriculum were compared to a cohort of interns exposed to their department’s independent within-discipline simulation education. Despite the absence of explicit curricular objectives or debriefing focus, it was noted during previous years’ IDSE curricula that one of the program’s implicit or “hidden curricula” was the potential improvement in intern interdisciplinary attitudes. Our study aimed to test the hypothesis that IDSE would positively affect intern between-discipline outlook and attitudes toward other medical disciplines based on pre-post questionnaire and focus group analysis. Additionally, factors affecting interdisciplinary attitudes, viewpoints, and prejudices, and their relationship to the simulation-based learning environment were explored.

## Methods

### Context

To investigate the effect of IDSE on interdisciplinary attitudes and viewpoints, the authors compared two cohorts of medical interns 6 months into their training at a tertiary care teaching facility: one cohort exposed to an existing interdisciplinary, longitudinal simulation-based curriculum and one cohort exposed to an existing independent within-discipline simulation-based curriculum. The post-graduate year one (PGY-1) Intern Simulation Common Critical Care Curriculum (4Cs) provides focused instruction on core critical care topics and procedural skills imperative for interns caring for adult patients [10]. This program aims to enhance and unify critical care education, with the primary goal of increasing confidence in managing common acute care scenarios, select procedural skills and communication skills. Included in this curriculum are important public health and system initiatives, including the Surviving Sepsis campaign, targeted temperature management in postcardiac arrest patients, and expeditious use of thrombolytics in acute ischemic stroke care. Additionally, advanced cardiac life support (ACLS)-based scenarios (ventricular arrest, asystole, pulseless electrical activity arrest) incorporate leadership and teamwork skills. Finally, ethical and spiritual topics, such as medical error disclosure and death notification, are integrated to enhance communication skills perceived as lacking in residency education.

The 4Cs annually educates approximately 60 interns. Small, interdisciplinary groups of learners from seven disciplines (internal medicine, family medicine, emergency medicine, general surgery, orthopedics, obstetrics/gynecology, and neurosurgery) attend this mandatory simulation-based curriculum, which represents the only formal longitudinal residency training within our hospital with multiple disciplines training together. Within this curriculum, each intern participated in three separate four-hour simulation-based sessions (Table 1) over a six-month period using Laerdal Sim Man 3G (Laerdal Medical AS, Stavanger, Norway). Four interns of mixed disciplines attended each session. Each scenario was led by one of the four interns, while the other three learners observed the scenario using real-time audio and visual feed. In the case of ACLS scenarios, the case was led by one intern with the other three learners participating as code members to add to the case fidelity. All scenarios ran approximately 10–15 min and were designed as acute floor emergencies with planned patient disposition to the ICU. A confederate nurse was used to facilitate each simulated scenario, and scenarios concluded with appropriate consultation to the ICU.

**Table 1:** Overview of 4Cs (Intern Simulation Common Critical Care Curriculum)

Session #1	
Ventricular Arrest/Post-Cardiac Arrest Care	
Anaphylaxis/Medication Error Disclosure	
GI Bleed/Consent/Central Venous Catheterization	
Surviving Sepsis Campaign	
Session #2	
Asystole/Death Notification	
Symptomatic Bradycardia/Transcutaneous Pacing	
Submassive Pulmonary Embolism	
Acute Coronary Syndrome/NSTEMI-ACS	
Session #3	
Unstable SVT/Synchronized Cardioversion	
Status Epilepticus/Lumbar Puncture/Video Laryngoscopy	
Cardiac Arrest due to Critical Hyperkalemia	
Cerebral Vascular Accident & Thrombolytics	

Immediately following each scenario, the scenario participant and three scenario observers underwent a shared debriefing. Debriefing lasted approximately 45 min and explored immediate reactions, course objectives, clinical management, pathophysiology, procedural instruction, and guided feedback to improve future clinical performance. Interdisciplinary attitudes, outlooks or stereotypes were not addressed in course objectives and never discussed in scenario debriefings. Debriefings were conducted by a dyad of four facilitators using the PEARLS model of debriefing [11] and consisted of the same predetermined learning objectives for all learners. Faculty dyads consisted of the principal investigator as a lead debriefer and one of three other faculty serving as an associate debriefer [12]. Each faculty had previous experience facilitating simulation debriefings and had co-debriefed in previous years’ curricula. The principal investigator led all co-debriefings to ensure all debriefings were uniform.

The comparison (non-IDSE) cohort, comprised of pediatric interns, was exposed to similar content in an independent longitudinal within-discipline simulation curriculum led by pediatric simulation faculty facilitators. The pediatric curriculum also included acute emergencies and specific procedural and communication skills during a similar number of small group simulation sessions over the investigation period.

### Study subjects and sampling

Involvement in this investigation was voluntary and was conducted from January to August 2017 at a 900-bed teaching hospital that serves as a tertiary referral center, trauma center, and regional branch campus of a medical school. Sixty 4Cs interns (IDSE cohort) and 12 pediatric interns (comparison non-IDSE cohort) were available for study enrollment. Two separate focus groups were conducted with volunteers selected from both cohorts upon completion of the IDSE curriculum. The IDSE focus group included a purposive sample of interns to obtain perspective from each of the seven disciplines participating in the curriculum. The comparison focus group enrolled a convenience sample of seven interns not involved in IDSE. Both IDSE and non-IDSE cohorts were composed of interns having completed medical school 6 months prior.

One hundred-dollar stipends were provided to each intern participating in the focus groups to compensate for time involved in the investigation. There was no compensation for voluntary survey completion. Focus group and survey enrollment processes were approved by our institutional review board for waiver of consent.

### Quantitative data sources and analyses

A novel survey with a focus on interdisciplinary viewpoints and attitudes was used to compare interns involved in IDSE and those who were not involved in IDSE. The survey was based on a 10-point Likert scale (Appendix 1) and was presented within-curriculum to all IDSE interns at pre- and post-curricular time intervals. Surveys were made available to non-IDSE interns electronically via hospital-wide email distribution using Survey Monkey (SurveyMonkey.com, Palo Alto, California) at times coinciding with pre- and post-IDSE surveys to account for time-effects in changes in interdisciplinary outlook and attitudes. While the survey was a de novo, novel instrument developed by the PI, its items have some face validity and are consistent with domains in other measures designed to evaluate staff perceptions of safety, satisfaction and engagement in the workplace [13].

In order to pair IDSE intern data, confidential, unique blinded identifiers were assigned to each IDSE intern by a study coordinator who administered and maintained a confidential process for survey data collection independent of the study investigators. Non-IDSE interns were also de-identified using unique identifiers, as well as electronic survey hyperlinks known only to the study coordinator. Attempts to capture all non-IDSE surveys were made by follow-up email sent by the study coordinator at weekly intervals.

Descriptive statistics, including response counts (percentages), means (standard deviations) and medians (interquartile ranges [IQR]) were reported as appropriate. Comparison of the pre-curricular survey responses between the IDSE and non-IDSE cohorts was performed with the Wilcoxon rank sum test. A two-sample t-test was used to analyze the differences between the IDSE and non-IDSE cohorts when comparing their respective pre-test and post-test score differences. Since the data for each cohort was not normally distributed, the investigators compared the change in score (post-pre) for each group to establish a normal distribution in the dataset.

Further statistical analysis was completed with a one-way Analysis of Covariance (ANCOVA) design to examine the post-intervention score differences between the control and intervention cohort after controlling for pre-test scores. The scores were analyzed as a multi-item scale of seven questions to compare the difference of the overall composite pre-intervention and post-intervention scores. To create a multi-item scale, the response from each question was totaled and the mean was computed for analysis. By adjusting for pre-test scores, this approach ensured that post-test differences were due to the intervention rather than differences at pre-test or pre-test variation.

Both analyses were completed to show the differences in each question’s response as well as the overall difference in pre-test and post-test scores for the IDSE and non-IDSE cohorts. All analyses were performed with SAS Enterprise Guide®, version 5.1 (SAS Institute, Cary, NC) and significance was set to *p* < 0.05.

### Qualitative data sources and analysis

A narrative-based case study methodology was implemented in the tradition of ethnographic approaches [14] to further explore the effects of IDSE on interdisciplinary attitudes. This case study was bound by the 4Cs duration and by three data sources: detailed observations, reflective field notes, and focus groups. Detailed observations regarding interns and their simulation-based interdisciplinary interactions were documented in field notes by curriculum faculty (MB, SF, CW, AH). These observations and reflective notes became study journals. These journals yielded reflections and initial analysis during the study period. Recognizing reflexivity and bracketing as important components to qualitative study, investigators were reminded to be explicit in their biases within their reflective journals, as well as in any interpretations.

The IDSE director (principal investigator), who has 10+ years of simulation debriefing experience, a MS-HPEd degree, and experience in qualitative study, led both focus groups. A structured guide with open-ended questions (Appendix 2) was used to guide the discussion of intern perspectives on simulation-based learning and its impact related to interdisciplinary viewpoints and attitudes for both IDSE and non-IDSE groups. Additional probing questions were used to clarify examples recalled from past experiences and other deeply held beliefs.

Qualitative data analysis was overseen by an experienced qualitative PhD researcher/educator (LR), who was not involved in the IDSE curriculum. Two researchers conducted the analysis (MB and LR). Qualitative data from the focus groups were recorded, professionally transcribed verbatim, and analyzed using both nVivo Software (QRS International, Melbourne, Australia) and manual techniques. The principal investigator (MB) reviewed each focus group transcription line by line to identify emergent codes pertaining to the study and based on previously published determinants of “interprofessionality” (which link interprofessional education and collaborative practice) [15]. Words and passages representing codes were underlined and listed in the margins of the transcripts. Coding decisions for this study were made explicit in a data dictionary for consistency. These codes were discussed among the researchers until a consensus was reached. The final codes were clustered and grouped into emergent themes in an iterative process. Member checking was accomplished through sharing of the resultant themes with interns via email and asking them to provide feedback regarding the accuracy of the results to ensure their perceptions were appropriately represented.

## Results

### Quantitative results

All sixty IDSE interns completed both pre- and post-curricular surveys. Two IDSE respondents filled out the same unique identifier within the survey and thus could not be reliably paired. As such, these data were removed from the dataset. Twelve of twelve possible non-IDSE interns completed both pre- and post- surveys.

When comparing pre-curricular survey responses for IDSE and non-IDSE interns, baseline responses demonstrated the IDSE cohort believed others viewed their medical discipline more negatively (*p* = 0.031) and the non-IDSE group believed other medical disciplines viewed their discipline more positively (*p* = 0.024). No other significant differences between intern groups were identified by baseline survey.

When comparing pre- and post-curricular survey responses for IDSE and non-IDSE cohorts independently, IDSE exposure significantly impacted mean scores for multiple survey responses related to interdisciplinary viewpoints and attitudes **(**Table 2**)**. Specifically, there were statistically significant post-curricular increases in the positive perceptions of: 1) comfort level of learning with others from different disciplines [*x̅* = 1.164; *p* < 0.0001], 2) extent to which different disciplines worked well with one another [*x̅* = 0.691; *p* = 0.0069], 3) rating that learning together improved collegiality between medical disciplines [*x̅* = 0.855; *p* = 0.0002], 4) views of other medical disciplines [*x̅* = 0.764; *p* = 0.0031], 5) respect from other disciplines within the hospital system [*x̅* = 0.436; *p* = 0.0312], and 6) beliefs that IDSE was beneficial to interdisciplinary interactions [*x̅* = 1.418; p < 0.0001]. The comparison cohort, exposed to only within-discipline simulation education, demonstrated no statistically significant change in response ratings **(**Table 2**)**.

**Table 2:** Comparison of the Pre and Post Scores collected on 10-point Likert scale (1 = low/very little; 10 = high/very much

	Interdisciplinary Simulation-based Education (IDSE) Group						Non-IDSE Group					
	Pre-test mean	Post-test mean	∆ (Mean)	SD	*P*-Value	CI	Pre-test mean	Post-test mean	∆ (Mean)	SD	*P*-Value	CI
I am comfortable when learning with interns of different disciplines.	7.491	8.655	1.164	1.80	< 0.0001*	(0.6762,1.6510)	6.833	6.833	0.000	1.54	1.000	(−0.9768, 0.9768)
I believe that other medical disciplines work well with my discipline.	7.754	8.448	0.691	1.82	0.0069*	(0.1977, 1.1841)	7.750	7.583	−0.167	1.12	0.615	(−0.8749. 0.5415)
Learning together with other disciplines improves collegial interactions.	8.281	9.103	0.855	1.58	0.0002*	(0.4274, 1.2817)	8.333	8.750	0.417	1.38	0.318	(−0.4595, 1.2928)
I have the opportunity in residency to learn side by side with other disciplines.	8.158	8.500	0.345	2.03	0.2123	(−0.2032, 0.8941)	8.333	8.000	−0.333	1.07	0.305	(−1.0151, 0.3485)
I believe that other disciplines view my discipline in a negative light.	5.375	5.281	−0.302	2.74	0.4254	(−1.0559, 0.4522)	3.500	4.333	0.833	1.95	0.166	(−0.4033, 2.0699)
I believe that other disciplines view my discipline in a positive light.	5.965	6.690	0.764	1.83	0.0031*	(0.2702, 1.2571)	7.333	6.750	− 0.583	1.08	0.089	(−1.2718, 0.1052)
I feel respected by residents in other disciplines within my hospital system.	7.596	8.034	0.436	1.46	0.0312*	(−1.7750, 0.0689)	7.333	6.917	−0.417	1.38	0.318	(−1.2928, 0.4595)
The 4Cs has created a venue that is beneficial to interdisciplinary interactions.	7.754	9.121	1.418	1.73	< 0.0001*	(0.981, 1.855)						

*significant values

A one-way ANCOVA test was conducted to compare the mean post-intervention viewpoint scores for each cohort, while controlling for mean pre-intervention test differences. There was a significant difference between the mean post-intervention scores (*p* = 0.0018) **(**Table 3**)**.

**Table 3:** Comparison of composite Pre and Post Scores using ANCOVA method

Group	Post-score total mean	Std. Error	*P*-value
IDSE	54.663	0.6572	0.0018*
Non-IDSE	49.654	1.3832	

*significant values

When comparing the mean pre-test and post-test differences between IDSE and non-IDSE cohorts **(**Table 4**)**, the IDSE cohort demonstrated statistically significant improvement ratings compared to the non-IDSE cohort in: the views of other medical disciplines (*p* = 0.02) and comfort in learning with other medical disciplines (*p* = 0.04).

**Table 4:** Comparison of ∆ between the non-IDSE & IDSE groups

Question	∆ (Mean) non-IDSE	∆ (Mean) IDSE	Difference	P-Value	Confidence Interval
I am comfortable when learning with interns of different disciplines.	0.000	1.16	1.16	0.04*	(−2.28, −0.04)
I believe that other medical disciplines work well with my discipline.	−0.17	0.69	0.52	0.12	(−1.96, 0.24)
Learning together with other disciplines improves collegial interactions.	0.42	0.85	0.44	0.38	(−1.42, 0.55)
I have the opportunity in residency to learn side by side with other disciplines.	−0.33	0.35	0.68	0.27	(−1.89, 0.53)
I believe that other disciplines view my discipline in a negative light.	0.83	−0.30	−1.14	0.18	(−0.54, 2.81)
I believe that other disciplines view my discipline in a positive light.	−0.58	0.76	1.35	0.02*	(−2.44, −0.25)
I feel respected by residents in other disciplines within my hospital system.	−0.42	0.44	0.85	0.07	(−1.78, 0.07)

*significant values

### Qualitative results

Qualitative data uncovered four major themes pertaining to IDSE (Table 5). It was apparent from the intern narratives that IDSE impacted relationship-building, and their capacity for improved interaction with and reliance on other disciplines. The experience helped to improve the understanding of one another’s disciplinary roles and the need to rely on one another. The interactions with other disciplines at a similar stage in their training allowed for sharing common experiences, empathic understanding and development of mutual trust, even friendship. For example, one IDSE intern commented “All of us have strengths and weaknesses … and if I can have a colleague that can help, and knows more, not only are they going to do the right thing for the patient, I am going to learn from them. When the tables flip, then I am going to teach them something and they are going to learn from me. I think that is how medicine is designed to work.”

**Table 5:** IDSE vs. Non-IDSE interns

Definition of Theme	IDSE	Non-IDSE
Relationship building Capacity for improved interaction with and reliance on other disciplines.	• Benefits from working with/going through something together • Development/creation of connections/relationships • Opportunities to acknowledge humility and vulnerability of all caregivers • Enhanced empathy and sympathy for colleagues in learning environment	• Lack of trust of other disciplines • Limited introspection and reflection on possible sources of disagreement or error • More value judgments placed on actions of others
Communication openness Ability to hear and acknowledge other clinical perspectives and viewpoints	• Increased opportunities to clarify viewpoints • Greater openness to disciplinary perspective • More cordial, congenial dialogue	• Frustration with misunderstandings during requesting and receiving disciplinary perspective
Attitudinal shifts Reduction in stereotyping and bias toward individuals in other services	• Increased respect for knowledge within discipline • Development of mutual trust and appreciation of different approaches to care	• Perceived lack of mutual interest, respect and appreciation for and from other disciplines in care planning • Perceived differences in quality of care provided
Enhanced learner experience More robust, interactive experience with outcomes related to improved interdisciplinary collaboration	• Efficient experience for diverse learners at similar training levels • Common learning objectives consistent with institutional goals • Enhanced interaction allowing for shared perspectives and discussion	• Focus on technical skills and practice for a single discipline • Reflections and debrief limited to single discipline perspective

IDSE also fostered improved communication openness, affording more cordial and “easier” interactions. Specifically, the IDSE focus group described feeling “at ease” in communicating with other disciplines with a belief of mutual trust and confidence in sharing perspectives with other disciplines. This seemed to melt some existing barriers related to traditional stereotypes and bias toward other services and improved the ability to acknowledge other clinical perspectives and viewpoints. One learner stated “[IDSE] builds comfort with the people I worked with from the other departments. You naturally feel more comfortable with them, because you know what they can and can’t do.”

Through the development of relationships and improved communication, IDSE appeared to produce attitudinal shifting allowing for the development of trust and mutual respect. Improvements in interdisciplinary attitudes were highlighted despite the acknowledged existence of environmental and cultural factors such as departmental siloes, system logistics, engrained history, superiors’ biases, and long work hours. Beyond simply putting a name to a face, the program humanized colleagues as people all working hard to achieve a common goal in providing care. One intern stated, “[IDSE] humbled everybody … and to see [the typically depicted] more arrogant specialties be so humble … in a group setting, changed my perspective on all of the specialties overall.” A second learner added “If you can trust someone; know their work ethic; … their strengths and weaknesses … it definitely helps a lot.” Finally, a third trainee concluded “… [IDSE] was a great way for us to pull back and not let the stereotypes and … all that stuff take over.”

IDSE improved the quality of the educational experience by providing opportunities for interactions with a true depiction of real-life interdisciplinary patient care. Designed to efficiently maximize education for a diversity of learners with common individual and institutional goals, the interdisciplinary simulation experience produced an enhanced learner experience while improving interdisciplinary collaboration. The benefits of IDSE were best described by one of our IDSE interns: “… it builds collegiality of [those] within your specialty and also … outside your specialty.” One of the IDSE interns even seemed to recognize the hypothesized implicit curriculum “… it was the unscripted curriculum that occurred as we were sitting in that room, talking about certain philosophies. We talked … we shared. That happens now with people I work with … I think they are more comfortable to ask about things because we worked together in 4Cs.”

The non-IDSE group had limited perspective on the potential benefits of IDSE. Non-IDSE interns maintained traditional interdisciplinary attitudes while also only viewing the potential impact of IDSE on the clinical aspects of patient care. One intern commented on the prevailing non-interdisciplinary cohort attitudes by saying: “There are stereotypes … and stereotypes are there for a reason.” However, they also expressed a perception that IDSE could potentially produce positive results to “facilitate better what we experience in the hospital,” provide clinical perspective on unique pathology “that we don’t necessarily experience,” and allow us to “better know what [other disciplines were] looking for … and [could] do to help the patient.”

## Discussion

Overall, this study demonstrated improved between-discipline viewpoints and attitudes for interns who experienced IDSE when compared to interns who did not experience IDSE. Quantitative analysis demonstrated measurable improvements in the feelings of respect and perceptions of between-discipline collaboration and collegiality. A one-way ANCOVA test was conducted to compare the mean post-intervention viewpoint scores or each cohort, while controlling for mean pre-intervention test differences indicating the IDSE training directly affected overall viewpoint scores. Comments from IDSE-exposed interns repeatedly highlighted the development of more trusting and respectful cross-discipline relationships as a result of contact with other departmental interns and interdisciplinary simulation-based debriefing. Furthermore, the qualitative analyses suggested that these improved perceptions arise from experiences that enhance relationship building and communication openness. This facilitated a shift in attitudes that added value to the IDSE learning experience making it “more representative of how it works in real life.”

In comparison, the non-IDSE cohort perspective suggested limited insight and value-added for any positive effects of IDSE on between-discipline attitudes without having the experience. While considered a potential benefit of IDSE for their learning and future practice, they tended to remain steadfast in their attitudes and beliefs about interdisciplinary attitudes and traditional discipline-specific stereotypes. These qualitative results were consistent with quantitative data demonstrating no change in interdisciplinary attitude response ratings for the non-IDSE cohort.

In order to optimize the benefits of IDSE, IDSE interns emphasized the interdisciplinary simulation-based education needed to be deliberate and focused. Future research should address the training level of staff involved; curricula representative of current workforce paradigms; the presence of an interactive forum allowing discussion of multiple perspectives; and a safe learning environment with passionate educators who model interdisciplinary collaboration.

This study also adds to our understanding of how IPE frameworks can be used to facilitate a positive impact on attitudes, viewpoints, and collegiality between disciplines and departments. IDSE provides experiential learning through interaction, reflection, reconciliation, and knowledge sharing. IDSE also provides a forum for learners to work in small, diverse interdisciplinary simulation-based learning teams and thus prompting individuals to think of each other as colleagues rather than representatives of competing disciplines. This concept as defined by D’Armour as “interprofessionality” is “the development of a cohesive practice between professionals from different disciplines” furthermore suggesting that training practitioners to be competent and collaborative is essential for positive outcomes to emerge in practice settings [15].

Others have suggested the use of Allport’s theoretical framework of Intergroup Contact Theory [16] when attempting to understand the impact of IPE on interprofessional relationships [17]. Allport’s theory proposes that to produce attitude change, learning environments and groups need necessary conditions beyond that of simply having a collection of interprofessional students. These additional conditions (learners of equal status, commonality in goals, support from authorities, and a cooperative forum [17]) are similar to qualitative findings noted by IDSE interns.

Similar to Allport’s Intergroup Contact Theory, interprofessionality is affected by factors such as: the learning context, educator beliefs, institutional support, curricula with shared common goals that specifically model and highlight mutual respect, trust, collaboration, communication, and an understanding of the various roles and responsibilities of different disciplines [15]. In retrospect, the authors noted that the 4Cs curriculum addressed necessary conditions to promote interprofessionality and between-group attitude change [16, 18], although this was not the explicit intention of the original program design. These two paradigms help explain why this specific IDSE curriculum could demonstrate an impact on interdisciplinary attitudes. All 4Cs interns were of an equal training level within one hospital system, shared common curricular goals, had intergroup cooperative discussions without competition, and had institutional support of both faculty and the hospital system’s division of medical education.

Having different disciplines learn together in the simulation environment is not sufficient to produce improved interprofessionality. It has been theorized that groups must understand others’ similarities, as well as their differences, to successfully affect prejudices and stereotypes [19]. Noteworthy in this study, the non-IDSE focus group repeatedly highlighted that negative interactions and attitudes of other disciplines towards their own were believed to be due to the fact they were of a different discipline: “… just because we are pediatrics.” The non-IDSE group rationalized these feelings, citing other disciplines have “different pathways of thinking” and simply could not understand their discipline because others “don’t carry out the same [patient] management.” Interestingly, this cohort felt dissimilar and somewhat isolated from other disciplines despite all interns having just completed medical school and all interns having regular clinical interactions with each other within the hospital setting.

Conversely, the IDSE group (also composed of members of disciplines that are quite clinically distinct from one another) appeared to more quickly identify similarities as well as differences with other disciplines’ which helped them to better value each other’s strengths and weaknesses. The more open viewpoints expressed in the IDSE group contrasted to the narrowly engrained perception about the different cultures of various medical disciplines within the non-IDSE cohort. This highlights the important effects that IDSE can generate to shift interdisciplinary viewpoints and attitudes particularly at the time of residency, since medical interns likely enter residency with beliefs, attitudes, and stereotypes already formed [15].

IDSE provides early opportunities to learn about, from, and with each other [4], which ultimately has the potential to improve team communication and patient care. Increasing specialization in medicine and health care requires new approaches to educating future professionals in all disciplines. Without new approaches, traditional discipline-specific attitudes and viewpoints will reinforce the siloes among disciplines and the challenging interdisciplinary relations that exist.

### Limitations

The limitations of this study include the fact that the non-IDSE cohort was only made up of one discipline, while the IDSE cohort had representation from seven different disciplines. This investigation was bound by two previously implemented curricula. For this reason, it was not feasible to add interns outside of pediatrics to the non-IDSE cohort which potentially affected the true representation of all interns without IDSE exposure. However, the baseline experience of all study participants was similar in that both cohorts included interns from various disciplines who regularly worked with one another during the first 6 months of their residency programs. The pediatric residency program, as a primary discipline, is not isolated from the other residency disciplines during clinical practice. As such, the lack of an interdisciplinary simulation-based educational program, such as the 4Cs, represented a key difference in the type of education and interaction between the non-IDSE and IDSE cohorts.

We were unable to expand the sample size or control for any factors involved in the independent non-IDSE longitudinal within-discipline simulation-based education. Although the overall effect size of the differences between IDSE and non-IDSE cohorts was relatively small, the true effect size may have been obscured because the comparison was not sufficiently powered. Furthermore, the implementation of the non-IDSE curriculum is not exact when compared to the 4Cs curriculum. However, the pediatric residency maintains a robust simulation-based curriculum similar in aim, facilitation, frequency, and overall session formatting based on acute pediatric emergencies. This program’s interns were also the most similar and only available comparison group of medical interns not involved in the IDSE curriculum. Finally, simulation was the educational modality used to observe effects of interdisciplinary education on interdisciplinary outlook and attitudes in this study. The investigators acknowledge that other educational modalities, strategies and learning experiences may have contributed or yielded similar results. Further research in this area is essential to evaluate the needs and opportunities for developing new educational approaches for medical education.

## Conclusion

Our data demonstrated significant improvements in interdisciplinary viewpoints and attitudes by IDSE interns towards colleagues in other disciplines when compared to interns not exposed to IDSE. Results reflect evidence that IDSE improves factors previously highlighted in current IPE frameworks on interprofessionality and intergroup attitude change [15, 16, 18]. Future IDSE should more purposefully focus on the need for integrating experiences that promote the development of skills in relationship building and communication openness while imparting basic clinical and procedural content. In a health system that has become increasingly specialized and siloed, this teaching strategy has the potential to reduce negative biases and prejudices, improve collegial interactions, and ultimately improve patient care. This realization will act as a future driver for more simulation-based training with an interdisciplinary focus within our hospital system.

## Data Availability

All datasets during and/or analyzed during this study are available from the corresponding author on reasonable request.

## Appendix 1

### Survey questions on interdisciplinary attitudes

Please rate your answers to the following on a scale from 1 to 10 (1 = low/very little, and 10 = high/very much)

1. I am comfortable when learning with other interns of different disciplines.
2. I believe that other medical disciplines work well with my discipline.
3. Learning together with other disciplines improves collegial interactions.
4. I have the opportunity in residency to learn side by side with other disciplines.
5. I believe that other disciplines view my discipline in a negative light.
6. I believe that other disciplines view my discipline in a positive light.
7. I feel respected by other residents in other disciplines within my hospital system.

## Appendix 2

### Semi- structured interview questions for focus groups

1. Tell me about your simulation experience to date.
  - How has simulation impacted your working relationships with other disciplines?
2. In general, how do you feel that (simulation) affects (has affected) your learning?
3. How do you feel that simulation (if made of multiple disciplines) might affect your interactions with peers from different disciplines?
  - How well do you believe that other medical disciplines work well with your discipline?
  - How does learning together with other disciplines improve collegial interactions?
  - What do you believe the benefit or lack of benefit is to learning with those in other disciplines?
4. What perceptions do you have about other disciplines within the hospital system?

- How other disciplines view your discipline? In a negative/positive light?
- How respected do you feel by other residents in other specialties within the hospital system?

5. What interaction do you have with other disciplines within our hospital? Outside rotations?

- What opportunities exist during residency to learn side by side with other disciplines?
- What is your level of comfort when learning with other interns of different disciplines?
- How has interdisciplinary training affected your learning and interactions with other departments/disciplines?

## Abbreviations

4Cs: Intern simulation common critical care curriculum
ACLS: Advanced cardiac life support
CI: Confidence interval
ICU: Intensive care unit
IDSE: Interdisciplinary simulation-based education
IPE: Interprofessional education
IQR: Interquartile ranges
PEARLS: Promoting excellence and reflective learning in simulation
PGY-1: Post-graduate year one
